# The burden of caring for adults with depression and suicidal ideation in five large European countries: analysis from the 2020 National Health and Wellness Survey

**DOI:** 10.1186/s12888-021-03511-9

**Published:** 2021-11-09

**Authors:** D. H. Jaffe, B. L. Balkaran, L. Yue, S. Mulhern-Haughey, J. Anjo

**Affiliations:** 1 Cerner Enviza, Tel Aviv, Israel; 2 Cerner Enviza, New York, NY USA; 3 Janssen Pharmaceuticals, Dublin, Ireland; 4 Janssen-Cilag Farmaceutica, Porto Salvo, Portugal

**Keywords:** Caregiver burden, Depression, Health-related quality of life, Healthcare resource utilization, Suicidal ideation, Work productivity and activity impairment

## Abstract

**Background:**

Suicidal ideation (SI) affects approximately 30–40% of those with major depressive disorder (MDD). To date, studies have examined the substantial humanistic and economic burden to caregivers of persons with MDD, however little is known of the impact of caring for persons with MDD when SI is present. This study examined the additional burden imposed on caregivers for persons with unipolar depression and SI in five major European countries.

**Methods:**

A retrospective, cross-sectional analysis was conducted in five European countries using 2020 Europe National Health Wellness Survey (France, Germany, Italy, Spain and the United Kingdom) to compare differences in health and economic outcomes between caregivers of adults with unipolar depression and SI (CAUD-SI) and caregivers of adults with unipolar depression without SI (CAUD-nSI). The outcomes assessed included health-related quality of life (HRQoL; Medical Outcomes Study Short Form [SF-12v2]), health status (Short-Form 6 Dimension [SF-6D], EuroQol 5-Dimension 5-Level [EQ-5D-5L]), Work productivity and activity impairment (WPAI) and healthcare resource utilization (HRU). Linear mixed models and generalized linear mixed models adjusted for covariates were used to compare the two groups on outcomes of interest.

**Results:**

Of 62,319 respondents, 0.89% (*n* = 554) were CAUD-SI and 1.34% (*n* = 837) were CAUD-nSI. In adjusted models, CAUD-SI reported greater humanistic burden than CAUD-nSI, with lower HRQoL (PCS: 42.7 vs. 45.0, *p* < 0.001 and MCS: 37.5 vs. 38.9, *p* = 0.007) and health status (SF-6D: 0.57 vs 0.60, *p* < 0.001 and EQ-5D-5L: 0.58 vs 0.66, *p* < 001). CAUD-SI respondents reported significantly higher economic burden than CAUD-nSI respondents for WPAI (percent activity impairment: 64.9% vs. 52.5%, *p* = 0.026) and HRU (provider visits: 10.0 vs. 7.9, *p* < 0.001, emergency room visits: 1.49 vs. 0.73, *p* < 0.001 and hospitalizations: 1.03 vs. 0.52, *p* < 0.001).

**Conclusion:**

In five European countries, caregivers of adults with depression and SI experience additional humanistic and economic burden than caregivers of adults with depression and no SI. Distinguishing caregiver groupings and their unique burden provide important insights for providing targeted support and interventions for both the patient and caregiver.

**Supplementary Information:**

The online version contains supplementary material available at 10.1186/s12888-021-03511-9.

## Background

Depression is a debilitating mental health condition that affects approximately 350 million people worldwide [1, 2]. Globally, 5.5% of all years lived with disability are attributed to depression [3]. In Europe, the lifetime prevalence of major depressive disorder (MDD) is estimated to be 11.3% [4]. It is the primary risk factor associated with suicide and suicidal ideation (SI) [5, 6] affecting 30–40% of those with MDD [7].

MDD and SI independently impose substantial humanistic burden defined and measured as the impact of the condition on health-related quality of life (HRQoL) and health status, and economic burden assessed using work productivity and medical and healthcare costs [8–14]. Moreover, the additional burden on persons with depression and SI was demonstrated in a recent cross-sectional survey of five European countries [13]. The consequences of these burdens and impairments include an increased dependency and reliance on long-term assistance for daily activities [15]. This resultant spillover effect [16] on informal caregivers can result in physical, emotional, social, and financial strain [17, 18].

The prevalence of informal caregivers in Europe is reported to be 34.3% while intensive caregiving, defined as providing care for a minimum of 11 h/week, is estimated at 7.6%; however, there is considerable variation in the prevalence of caregivers between countries [19–21]. In general, caregivers, regardless of their time investment, experience higher physical and psychological complications than non-caregivers [21]. Among caregivers, the humanistic and economic burden varies and is dependent on factors including the patient’s disease [21–24] and its severity [25]. For example, caregivers of patients with psychiatric disorders experience higher rates of anxiety and depression compared to caregivers of patients with physical illness [21, 24]. Caregivers of adults with depression have approximately three times more frequent emergency room visits or hospitalizations compared to non-caregivers and 20% more healthcare resource utilization (HRU) than caregivers of adults with other chronic diseases [26]. An inverse relationship has been observed between disease severity and caregiver burden leading to reduced work participation, greater HRU use, and higher informal caregiving costs [25].

Clearly, a one-size-fits-all caregiver profile and burden is too simplistic for research, policy, and care implications [27, 28]. To date, studies related to depression caregiving have primarily focused on MDD and its impact on caregiver outcomes in relation to MDD severity; these studies did not account for the additional caregiver burden associated with SI. Further, high rates of SI among patients with depression in Europe necessitates a comprehensive understanding of the compounded burden of depression and SI on caregivers. The aim of this study was to assess the additional humanistic and economic burden experienced by persons caring for patients with depression and SI in five major European countries.

## Methods

### Study population

#### Data source

Data for this large, nationally representative, retrospective observational study were extracted from Kantar Health’s 2020 Europe National Health and Wellness Survey (NHWS). The 2020 Europe NHWS is a self-administered, web-based survey of adults aged ≥18 years (*N* = 62,319) in five European countries – France, Germany, Italy, Spain, and the United Kingdom (UK). Respondents are identified through opt-in online survey panels using quota sampling by sex and age to create a demographically representative sample of the adult population in each country. To further ensure accurate representation of the survey, respondents may be recruited via telephone recruitment or invited to complete an online survey at private computer centers. The 2020 Europe NHWS received exemption from the Pearl Independent Review Board (IRB) in accordance with FDA 21 CFR 56.104 and DHHS 45 CFR 46.104 regulations (19-KANT-204). All survey data collected were anonymous.

### Study sample

Respondents included in the current study were aged ≥18 years, could read and write in the primary language of the country in which the study was conducted, and provided informed consent. Using the overall 2020 Europe NHWS sample of 62,319 respondents, we identified caregivers of adults with unipolar depression based on affirmative response to the survey query “Are you currently caring for an adult relative with any of the following conditions = depression?” Excluded were those who reported caring for an adult relative with bipolar disease or schizophrenia, since these patients have different underlying psychopathological factors even though their patterns of depressive episodes may be similar to adults with unipolar depression [29, 30]. The final sample cohort was grouped according to their response to “Has the person you care for with depression had thoughts that they would be better off dead or of hurting themselves in some way or have planned or attempted to end their life /commit suicide in the past six months?” into two categories: caregivers of persons with unipolar depression and SI (positive response) and caregivers of adults with unipolar depression but no SI (negative response) (see Table 1).

**Table 1:** Sociodemographic and health characteristics of caregivers of unipolar depression + SI and caregivers of unipolar depression + no SI

Characteristics	Caregivers of unipolar depression + SI (*N* = 554)	Caregivers of unipolar depression + no SI (*N* = 837)	*p* value^a^
**Socio-demographic characteristics**			
**Age (years), mean ± SD**	40.7 **±** 15.2	43.5 **±** 15.2	**< 0.001**
** Gender, n (%)**			
Female	298 (53.8)	487 (58.2)	0.106
Male	256 (46.2)	350 (41.8)	
** Marital Status, n (%)**			
Married or living with partner	362 (65.5)	540 (64.9)	0.831
Not Married	191 (34.5)	292 (35.1)	
** Education, n (%)**			
University degree	207 (37.8)	270 (32.5)	**0.040**
Less than university degree	340 (62.2)	562 (67.5)	
** Annual Household Income, n (%)**			
Less than €49,999 or £39,999	409 (76.9)	599 (76.6)	0.906
€50,000 or £40,000 or more	123 (23.1)	183 (23.4)	
** Employment Status, n (%)**			
Employed	382 (69.0)	513 (61.3)	**0.014**
Not employed	113 (20.4)	211 (25.2)	
Retired	59 (10.6)	113 (13.5)	
**Health characteristics**			
**Body Mass Index, n (%)**			0.137
Underweight (< 18.5 kg/m^2^)	33 (6.0)	30 (3.6)	
Normal weight (18.5 to < 25.0 kg/m^2^)	217 (39.2)	330 (39.4)	
Overweight (25.0 to < 30.0 kg/m^2^)	154 (27.8)	266 (31.8)	
Obese (30.0 kg/m^2^ and above)	109 (19.7)	161 (19.2)	
Decline to answer	41 (7.4)	50 (6.0)	
**BMI, mean ± SD (valid n)**	26.3 ± 7.0 (*n* = 513)	26.4 ± 6.3 (*n* = 787)	0.684
**Smoking Status, n (%)**			**< 0.001**
Current	244 (44.0)	276 (33.0)	
Former	118 (21.3)	194 (23.2)	
Never	192 (34.7)	367 (43.8)	
**Alcohol use, n (%)**			0.188
Currently consume	439 (79.2)	638 (76.2)	
Currently do not consume	115 (20.8)	199 (23.8)	
**Exercise,**^**b**^** n (%)**	421 (76)	571 (68.2)	**0.002**
**CCI Categories, n (%)**			0.161
0	368 (66.4)	594 (71.0)	
1	106 (19.1)	146 (17.4)	
2+	80 (14.4)	97 (11.6)	
**CCI, mean ± SD**	0.71 **±** 1.47	0.53 **±** 1.16	**0.012**
**Self-reported medical diagnosis of anxiety, n (%)**			0.141
Yes experienced + No diagnosis	84 (15.2)	137 (16.4)	
Yes experienced + Yes diagnosis	188 (33.9)	249 (29.7)	
Did not experience	282 (50.9)	451 (53.9)	
**Self-reported medical diagnosis of depression, n (%)**			**< 0.001**
Yes experienced + No diagnosis	125 (22.6)	155 (18.5)	
Yes experienced + Yes diagnosis	265 (47.8)	314 (37.5)	
Did not experience	164 (29.6)	368 (44.0)	
**Self-reported medical diagnosis of sleep problems, n (%)**			**0.018**
Yes experienced + No diagnosis	148 (26.7)	205 (24.5)	
Yes experienced + Yes diagnosis	82 (14.8)	91 (10.9)	
Did not experience	324 (58.5)	541 (64.6)	
**PHQ- 9 score, n (%)**			**< 0.001**
Minimal, 0–4	85 (15.3)	213 (25.4)	
Mild, 5–9	112 (20.2)	282 (33.7)	
Moderate, 10–14	156 (28.2)	180 (21.5)	
Moderately severe, 15–19	116 (20.9)	106 (12.7)	
Severe, 20–27	85 (15.3)	56 (6.7)	
**PHQ- 9 score, mean ± SD**	12.3 **±** 6.8	9.1 **±** 6.3	**< 0.001**

*Note: CCI* Charlson comorbidity index, *PHQ-9* Patient Health Questionnaire (9 questions), *SI* Suicidal ideation

^a^Statistical testing for categorical variables were calculated using Chi-square test and for continuous variables using ANOVA, with statistically significant values in bold

^b^Exercise was defined as yes/no reporting exercising for 20 min or more at least on one occasion in the past 30 days

### Measures

#### Sociodemographic and health characteristics

Demographic measures included age, gender (male or female), marital status (married/living with partner or not married), education (university degree or less than university degree), household income (more than €50,000/£40,000 or less than €49,999/£39,999) and employment status (employed, not employed or retired).

Health characteristics examined were body mass index (BMI) (underweight [< 18.5 kg/m^2^], normal weight [18.5 to < 25.0 kg/m^2^], overweight [25.0 to < 30.0 kg/m^2^], obese [≥30.0 kg/m^2^], exercise at least 20 min on at least one occasion in the past 30 days (no or yes), alcohol consumption (consume or does not consume) and smoking status (current, former, or never). Burden of non-psychiatric comorbidities was estimated using the Charlson Comorbidity Index (CCI) [31]. The CCI index is a function of health condition and summation of overall result obtained in the survey questionnaire that assess disease experience (“Which of the following conditions have you ever experienced?”). Higher total index score implies greater comorbidities. Mental health related conditions, anxiety, depression, and sleep difficulties were categorized according as those experiencing the condition with or without a diagnosis. In addition, the depression screening Patient Health Questionnaire (PHQ-9) score was used to characterize respondents depressive symptoms as minimal (0–4), mild (5–9), moderate (10–14), moderately severe (15–19), and severe (20–27).

#### Humanistic and economic outcomes

HRQoL was determined using the Medical Outcomes Study 12-Item Short-Form Health Survey version 2 (SF-12v2) [32, 33], a multipurpose, generic health status instrument. Respondents are asked 12 questions based on the past four weeks. These questions are designed to map eight different health domains:

- Physical functioning – limitations in moderate physical activity
- Physical role functioning – accomplishes less due to physical health
- Bodily pain – pain interferences with work (in and out of home)
- General health – general health rating
- Social role functioning – interference of physical health or emotional problems with social activities
- Emotional role functioning – accomplishes less due to emotional problems
- Vitality – energy and fatigue
- Mental health – psychological distress and well-being

Two component summary scores assessed were physical component summary (PCS) and mental component summary (MCS) scores. Health domain scores are normally distributed over a range of 0 to 100, mean average of summary score equals 50 with a standard deviation (SD) of 10 for the US general population. Scores can be interpreted based on standard US derived scoring algorithm or country specific scoring or comparison groups of interest. Higher scores for each domain and summary score reflect better quality of life. A minimal clinically important difference (MCID) for the MCS and PCS is considered 3.0 [34].

Short-Form 6 Dimension (SF-6D) derived from SF-12v2 (prior 4 weeks) [35] and the EuroQol 5-Dimension 5-Level version (EQ-5D-5L) (current health status, i.e., ‘today’) [36, 37]) were used to assess health status. In general, health status measures are normally distributed and range between 0 (health state equivalent to death) to 1 (health state equivalent to perfect health). Scoring of the SF-6D considers 6 of the 8 SF-12 domains, including physical functioning, physical role functioning, emotional functioning, social functioning, bodily pain, mental health and vitality. The EQ-5D-5L consists of 5 dimensions: mobility, self-care, usual activities, pain/discomfort, and anxiety/depression often used to evaluate quality-adjusted life years associated with a health state [38]. An MCID of 0.07 is considered for the EQ-5D-5L and 0.04 for the SF-6D [39].

Work productivity loss and activity impairment due to ill health for employed (full-time, part-time or self-employed) respondents was assessed using the Work Productivity and Activity Impairment questionnaire (WPAI) [40]. It is a six-item validated instrument that measures the aspects of work productivity based on four subscales: absenteeism (% of work time missed due to health problems in the past seven days), presenteeism (% impairment at work due to health problems in the past seven days), overall work productivity loss metric (overall % estimate combining absenteeism and presenteeism in the past seven days), and activity impairment (% impairment in non-work activities due to one’s health in the past seven days). Higher percentage value for each of the subscale correspond to greater impairment.

HRU data were defined as frequency of visits to various health care providers (HCPs) in the past six months. These include traditional providers (e.g., general practitioners, nurses) and specialist visits (psychiatrist or psychologist/therapist), emergency room (ER) visits, as well as hospitalizations.

### Statistical analysis

The study compared the characteristics and outcomes of caregivers of unipolar depression + SI (CAUD-SI) and caregivers of unipolar depression + no SI (CAUD-nSI). Descriptive statistics included means and SDs for continuous variables and frequencies and percentages for categorical variables. Missing data is minimized by allowing respondents the option of ‘decline to answer’ or ‘prefer not to answer’ for items asking about potentially sensitive topics, such as weight or income. The resultant small amount of missing data are expected to be missing at random (MAR).

Bivariate analyses evaluated differences between caregiver groups using ANOVA for continuous variable and chi-square tests for categorical variables. Multivariate analyses were employed to examine the association between caregiver group and outcome after adjusting for potential confounding factors. Model choices were determined according to the distribution of each outcome. Linear mixed models with gaussian distribution were used for health outcomes (EQ-5D-5L, SF-6D, and SF-12v2 scores) and generalized linear mixed models with negative binomial distribution were used for economic outcomes (WPAI metrics, healthcare resource utilization) after controlling for the confounding effect of sociodemographic (age, education, employment status) and health status (smoking status, exercise, CCI) factors using backward models. Adjusted means, standard errors (SE), regression coefficients, *p*-values, and 95% confidence intervals were calculated for all the dependent variables. *P* < 0.05 was considered as statistically significant.

All statistical analyses were conducted using SPSS v23.0, SAS v9.4, and/or R v3.6.0 or higher.

## Results

### Caregiver characteristics

**Fig. 1:**
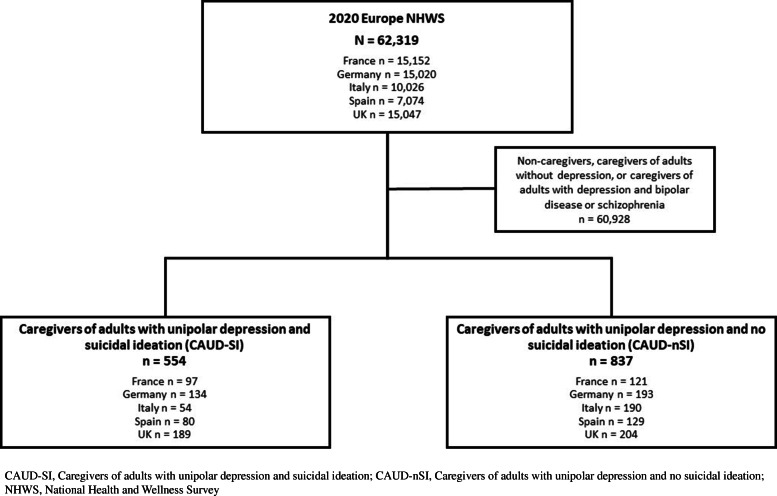
Study population from five European countries: 2020 NHWS

A total of 62,319 respondents (France *N* = 15,152; Germany *N* = 15,020; Italy *N* = 10,026; Spain *N* = 7074; UK *N* = 15,047) were included in the study. Of these respondents, 554 (0.89%) were identified as CAUD-SI whereas 837 respondents (1.34%) were identified as CAUD-nSI (Fig. 1).

Unadjusted comparisons showed that all caregivers, regardless of subgroup, were more often women (CAUD-SI = 53.8% vs. CAUD-nSI = 58.2%, *p* = 0.106), married (CAUD-SI = 65.5% vs. CAUD-nSI = 64.9%, *p* = 0.831), and reported lower annual household income (CAUD-SI = 76.9% vs. CAUD-nSI = 76.6%, *p* = 0.906) **(**Table 1). Differences between groups were observed with CAUD-SI respondents being younger (40.7 ± 15.2 years vs. 43.5 ± 15.2 years, *p* < 0.001), more educated (university degree: 37.8% vs. 32.5%, *p* = 0.040) and more likely to be employed (69.0% vs. 61.3%, *p* = 0.014) compared to CAUD-nSI respondents, respectively. CAUD-SI respondents were more likely to smoke (44.0% vs. 33.0%, overall *p* < 0.001) and exercise (76.0% vs. 68.2%, *p* = 0.002) and had a higher average CCI score (0.71 vs. 0.53, *p* = 0.012).

CAUD-SI respondents reported higher mental health burden in terms of self-reported medical diagnosis (SR-MD) of depression (47.8% vs. 37.5%, overall distribution *p* < 0.001) and sleep problems (14.8% vs. 10.9%, overall distribution *p <* 0.05) (Table 1). Additionally, differences in PHQ-9 scores were noted to be significant between CAUD-SI and CAUD-nSI (*p* < 0.001), with higher proportion of CAUD-SI respondents reporting moderate, moderately severe, and severe depression.

### Unadjusted comparison of outcome for CAUD-SI and CAUD-nSI

Unadjusted analyses of outcomes related to caregiving for depression with and without SI indicated substantially higher burden among CAUD-SI respondents compared with CAUD-nSI (Table S1).

CAUD-SI respondents reported significantly lower scores for each domain – physical functioning (44.9 vs. 48.7, *p* < 0.001), bodily pain (41.8 vs. 44.3, *p* < 0.001), physical role functioning (40.4 vs. 43.7, *p* < 0.001), emotional role functioning (34.2 vs 38.7, *p* < 0.001), and social role functioning (38.1 vs. 41.5, *p* < 0.001), as well as lower PCS (46.2 vs. 48.5, *p* < 0.001) and MCS scores (38.4 vs. 40.3, *p* < 0.001).

CAUD-SI respondents also reported lower health status as measured by EQ-5D-5L (0.63 vs. 0.73, *p* < 0.001) and SF-6D health utilities (0.60 vs. 0.64, *p* < 0.001) than CAUD-nSI.

In terms of WPAI, CAUD-SI respondents reported higher levels of absenteeism (24.1 vs. 14.6, *p* < 0.001), presenteeism (48.4 vs. 34.7, *p* < 0.001), overall work productivity loss (54.7 vs. 39.6, *p* < 0.001), and activity impairment (49.0 vs. 39.5, *p* < 0.001) compared with CAUD-nSI respondents.

When assessing HRU, a higher percentage of CAUD-SI respondents reported visit to a psychologists or therapists in the past six months (12.3% vs. 8.5%, *p* = 0.021), however no difference was noted for visits to a psychiatrist (11.2% vs. 8.6%, *p* = 0.109) compared with CAUD-nSI respondents. Further, significantly higher traditional provider visits (7.9 vs. 6.5, *p* = 0.005), ER visits (1.48 vs. 0.59, *p* < 0.001), and hospitalizations (0.83 vs. 0.32, *p* < 0.001) were observed in CAUD-nSI respondents than CAUD-nSI.

### Adjusted comparison of outcomes for CAUD-SI and CAUD-nSI

**Fig. 2:**
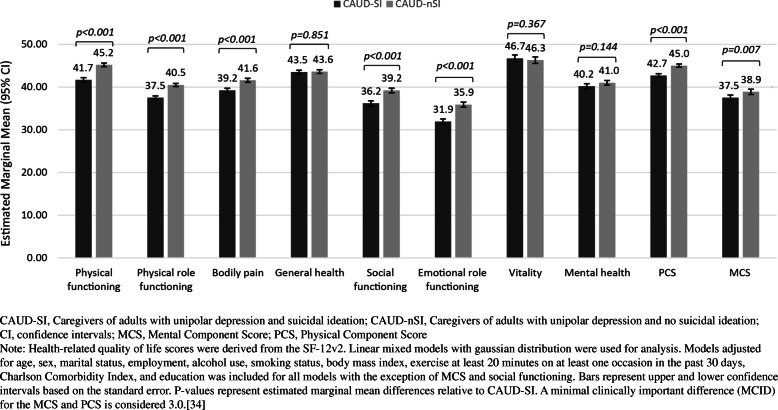
Adjusted marginal means for health-related quality of life of caregivers of unipolar depression + SI and caregivers of unipolar depression + no SI

Multivariable models adjusted after controlling for covariates showed that CAUD-SI versus CAUD-nSI had substantially lower adjusted means for MCS (37.5 vs. 38.9, *p =* 0.007) and PCS (42.7 vs. 45.0, *p* < 0.001) (Table S2, Fig. 2). Specific domains of the SF-12v2 with statistically significant marginal mean differences between groups included physical functioning (difference = 3.5), bodily pain (difference = 2.4), physical role functioning (difference = 3.0), emotional role functioning (difference = 4.0), and social role functioning (difference = 3.0).

**Fig. 3:**
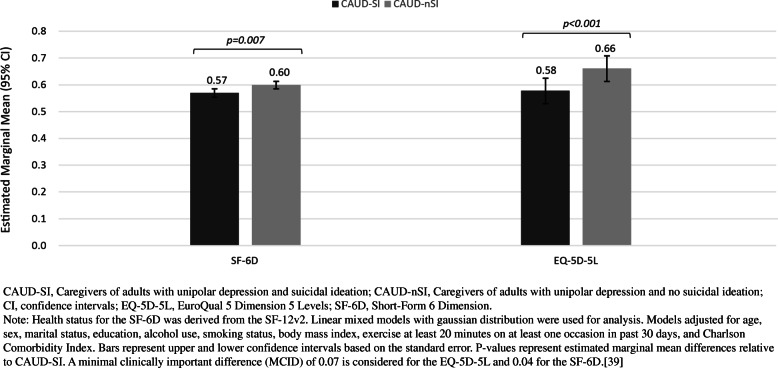
Adjusted marginal means for health status of caregivers comparing unipolar depression + SI and caregivers of unipolar depression + no SI

In the adjusted model, the CAUD-SI group had significantly poorer health status compared to CAUD-nSI for both the SF-6D (0.57 vs 0.60, *p* < 0.001) and EQ-5D-5L (0.58 vs 0.66, *p* < 001). The MCID of > 0.07 points was observed for the EQ-5D-5L (Table S2, Fig. 3).

**Fig. 4:**
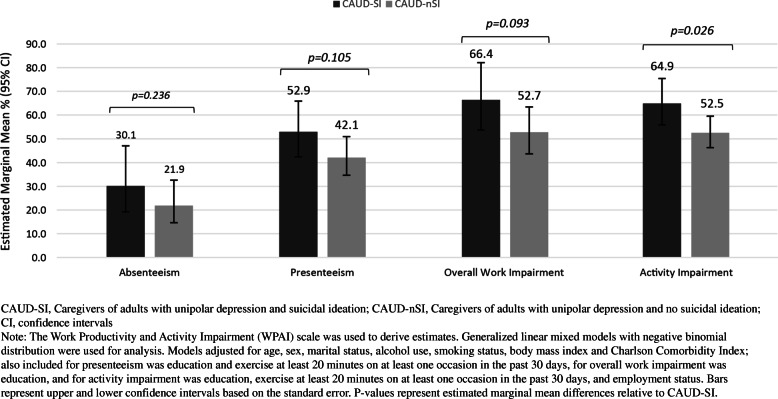
Adjusted marginal means for work productivity and activity impairment of caregivers comparing unipolar depression + SI and caregivers of unipolar depression + no SI

Comparison of WPAI in adjusted models showed no significant differences in absenteeism, presenteeism, or overall work impairment between CAUD-SI and CAUD-nSI respondents (*p* > 0.05), but differences in activity impairment were observed between the two groups (*p* = 0.026). (Table S2, Fig. 4).

**Fig. 5:**
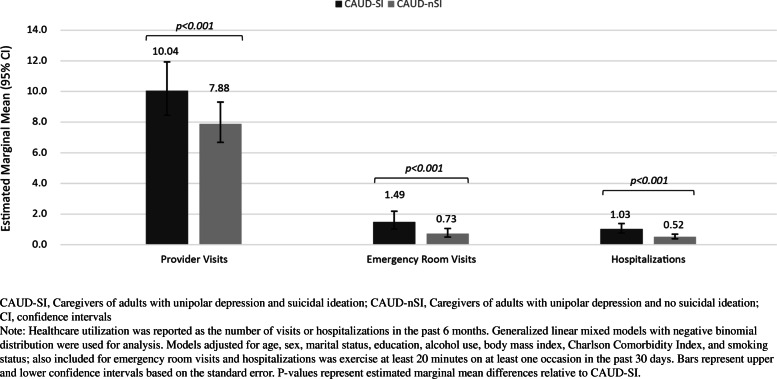
Adjusted means for healthcare resource use in past  months comparing caregivers of unipolar depression + SI and caregivers of unipolar depression + no SI CAUD-SI

CAUD-SI respondents reported higher estimated marginal means for provider visits (10.0 vs. 7.9, *p* < 0.001), ER visits (1.49 vs. 0.73, *p* < 0.001), and hospitalizations (1.03 vs. 0.52, *p* < 0.001) compared with CAUD-nSI respondents (Table S2, Fig. 5).

## Discussion

The present study reports the additional burden experienced by caregivers of adults with unipolar depression due to presence of SI across five European countries. We show that when compared to those caring for adults with unipolar depression and no SI, caregivers of adults with unipolar depression and SI experience a substantial excess humanistic and economic burden, with lower health status and HRQoL, and increased HRU. This observation extends our previous work by showing the increased burden of caregiving for adults with depression compared to other diseases [26].

Factors associated with caregiver burden include both caregiver and patient characteristics [21–25, 27, 28, 41–43]. For example, caregiver characteristics associated with poor outcomes include age, gender, socioeconomic status, levels of informal support, comorbidities, and choice of being a caregiver [21, 41, 43]. Patient characteristics affecting caregiver burden include, relationship to the carer, type, severity, and level of disability of disease [27, 28]. A meta-analysis examining the effects of caregiving on physical health showed that the patient’s overall impairment and cognitive complications in addition to the caregiver’s involvement (number of caregiving hours per day/per week and caregiving tasks, overall duration of caregiving), physical health, and available social support impact the degree of caregiving burden [41, 43].

In this study, demographic characteristics of caregivers of adults with depression are consistent with previous report, with a little over half of the respondents being female [44] and two-thirds married or living with their partner and employed [45]. Interestingly, characteristics of caregivers for unipolar depression and SI differed significantly from those without SI by age, employment, smoking status, CCI, and mental health (diagnosis and/or experience) – anxiety, depression, and sleep issues. Differences in socio-demographic and health characteristics may be predictors (e.g., age, employment) or outcomes (e.g., anxiety, depression) associated with being a caregiver for patients with depression and SI. These findings reflect a critical distinction between caregiver groups with likely different needs [27, 28].

The impact of SI on the caregiver of patients with depression is most prominent in the functional domains and for the domain related to bodily pain of the SF-12v2, whereby an MCID was present for the physical, emotional role, and social functional scores. In contrast, no statistically significant group differences were observed for general health, mental health, or vitality. Furthermore, caregivers of patients with unipolar depression and SI reported greater impairment of health status for both utility scores; however, an MCID was observed for the EQ-5D-5L and not the SF-6D. These findings suggest that the additional burden of caring for depression with SI is likely related to more practical limitations (EQ-5D) and less to a general sense of well-being (SF-6D) [46].

The negative impact of caregiving on work participation, perhaps another indicator of a functional domain, is a notable finding as it highlights the patient’s burden outside of the family space and into the workplace and society [25]. Recent studies of caregivers of chronic illness and specifically depression reported greater work and overall activity impairment, with higher levels of absenteeism and presenteeism and related costs compared with non-caregivers [25, 26, 47]. In this study, the additional work-related burden among employed caregivers was not observed in fully adjusted models. However, overall activity impairment was substantially higher among those caring for depression and SI than depression alone, which are consistent with the functional limitations observed for HRQoL measures.

Higher HRU (provider visits, ER visits, hospitalization) were reported for those caring for adults with unipolar depression and SI compared to those without SI. In a similar cross-sectional study in Europe, of patients with unipolar depression with and without SI, higher HRU was observed in patients with unipolar depression and SI compared to those with unipolar depression without SI [13]. As patients with more severe depression have higher overall rates of hospitalization and specifically SI-related hospitalizations, it is likely that caregivers’ heightened physical and psychological stresses lead to negative health outcomes requiring care.

### Strengths and limitations

The strengths of the study include self-reported outcomes that are uniformly derived across all five European countries. The sample of caregivers is drawn from a representative sample of the general population of each country in which the study was conducted, which notably differs from many caregiver studies that use convenience samples [27, 43]. Recall bias is minimized since most survey questions are based on current practice and attitude. Also, missing data is minimized by including ‘don’t know’ or ‘decline to answer’ as options for items regarding potentially sensitive topics, such as weight or income.

The present study is not without limitations. The NHWS is a panel-based survey and may not reach segments of populations including those with limited internet access, disabilities, or lack of familiarity with online surveys. Further, caregivers who can read and write in the language of their resident country were included in the NHWS, thereby excluding potential caregivers who do not meet this criteria; although we would assume this potential bias to be minimal as most caregivers of their adult relatives are likely fluent in their country of residence’s language. The study relies on self-reported data and therefore diagnosis of depression and other relevant diagnoses (e.g., those used in the comorbidity index) and report of healthcare visits cannot be confirmed. Also, since only measured variables were accounted for in regression analysis, there is possibility of groups differing on unmeasured variables that may have an impact on outcomes. Finally, the NHWS does not capture caregiving for a patient based on patient characteristics such as severity, medication use, or duration of their disease. This in turn, is likely to affect the assessment of burden as caregiving is impacted by degree of severity of disease and depression is a complex disorder that is modulated by several factors including severity, duration and overall management of disease.

## Conclusions

The present study shows that in these five European countries, caregivers of adults with depression and SI compared to caregivers of adults with depression without SI experience an additional humanistic and economic burden. It is important to critically assess each unique caregiver group and their associated burden in order to estimate burden as well as provide targeted support and interventions for both the patient and caregiver.

## Supplementary Information

**Additional file 1.**

## Data Availability

NHWS data used in this study are available for noncommercial research and validation purposes, upon request. Interested individuals may access the data for the purposes above in the same manner as the authors did without any additional restrictions. Interested parties should contact the corresponding author on reasonable request.

## Abbreviations

BMI: Body mass index
CAUD-nSI: Caregivers of unipolar depression + no SI
CAUD-SI: Caregivers of unipolar depression + SI
CCI: Charlson Comorbidity Index
ER: Emergency room
EQ-5D-5L: EuroQol 5-Dimension 5-Level
HCP: Health care provider
HRU: Healthcare resource utilization
HRQoL: Health-related quality of life
IRB: Independent review board
MDD: Major depressive disorder
MCS: Mental component summary
MCID: Minimal clinically important difference
MAR: Missing at random
NHWS: National Health and Wellness Survey
PHQ: Patient health questionnaire
PCS: Physical component summary
SD: Standard deviation
SE: Standard error
SF-12v2: Short-Form Health Survey Version 2
SF-6D: Short-Form 6 Dimension
SI: Suicidal ideation
SR-MD: Self-reported medical diagnosis
UK: United Kingdom
WPAI: Work productivity and activity impairment questionnaire
