# Supplementary material for: The burden of caring for adults with depression and suicidal ideation in five large European countries: analysis from the 2020 National Health and Wellness Survey
Source: BMC Psychiatry. 2021 Nov 9;21:550. doi: 10.1186/s12888-021-03511-9 (PMC8576941; doi:10.1186/s12888-021-03511-9)
Supplement: Supplementary file 1 — Additional file 1. [file 12888_2021_3511_MOESM1_ESM.docx]

**Supplemental Table 1: Unadjusted comparisons of caregivers of unipolar depression + SI and caregivers of unipolar depression + no SI**

|  | Caregivers of unipolar depression + SI  (N=554) | Caregivers of unipolar depression + no SI  (N=837) | *p* value^a^ |
| --- | --- | --- | --- |
| HRQoL, mean ± SD | | | |
| Physical functioning | 44.9 **±** 11.0 | 48.7 **±** 9.7 | **<0.001** |
| Physical role functioning | 40.4 **±** 10.0 | 43.7 **±** 9.5 | **<0.001** |
| Bodily pain | 41.8 **±** 10.5 | 44.3 **±** 9.9 | **<0.001** |
| General health | 47.1 ± 11.1 | 46.8 ± 10.2 | 0.510 |
| Social role functioning | 38.1 **±** 9.7 | 41.5 **±** 9.3 | **<0.001** |
| Emotional role functioning | 34.2 **±** 12.0 | 38.7 **±** 11.8 | **<0.001** |
| Vitality | 48.5 **±** 10.6 | 48.1 **±** 9.4 | 0.528 |
| Mental health | 41.1 **±** 9.3 | 42.2 **±** 9.4 | **0.031** |
| PCS | 46.2 **±** 9.3 | 48.5 **±** 8.9 | **<0.001** |
| MCS | 38.4 **±** 9.4 | 40.3 **±** 9.6 | **<0.001** |
| Health Status, mean ± SD | | | |
| SF-6D | 0.60 ± 0.11 | 0.64 ± 0.11 | **<0.001** |
| EQ-5D-5L | 0.63 ± 0.27 | 0.73 ± 0.24 | **<0.001** |
| Work productivity and activity impairment (WPAI), mean % ± SD | | | |
| Absenteeism (employed only)^b^ | 24.1 **±** 28.5 | 14.6 **±** 24.0 | **<0.001** |
| Presenteeism (employed only)^c^ | 48.4 **±** 29.2 | 34.7 **±** 28.5 | **<0.001** |
| Overall work productivity loss (employed only) ^d^ | 54.7 **±** 31.5 | 39.6 **±** 31.4 | **<0.001** |
| Activity Impairment, mean ± SD | 49.0 **±** 28.3 | 39.5 **±** 28.9 | **<0.001** |
| Healthcare Resource Use (past 6 months) | | | |
| Specialist visits – psychiatrist, n (%) | 62 (11.2) | 72 (8.6) | 0.109 |
| Specialist visits – psychologist / therapist, n (%) | 68 (12.3) | 71 (8.5) | **0.021** |
| HCP visits, mean ± SD | 7.9 **±** 11.9 | 6.5 **±** 7.7 | **0.005** |
| ER visits, mean ± SD | 1.48 **±** 4.32 | 0.59 **±** 1.48 | **<0.001** |
| Hospitalizations, mean ± SD | 0.83 **±** 1.99 | 0.32 **±** 1.33 | **<0.001** |

*Note:* EQ-5D-5L = EuroQol 5-Dimension Health Questionnaire; HRQoL = health-related quality of life; MCS = Mental Component Score; PCS = Physical Component Score; SD = standard deviation; SF-6D = Short Form 6 Dimension; SF-12v2 = Revised Short Form 12 item Health Survey; SI = suicidal ideation

^a^ Statistical testing was performed using ANOVA and statistically significant values bolded.

^b^ Sample included caregivers of unipolar depression + SI *n* = 363 and caregivers of unipolar depression + no SI *n* = 490.

^c^ Sample included caregivers of unipolar depression + SI *n* = 359 and caregivers of unipolar depression + no SI *n* = 483.

^d^ Sample included caregivers of unipolar depression + SI *n* = 349 and caregivers of unipolar depression + no SI *n* = 475.

**Supplemental Table 2: Adjusted comparisons of caregivers of unipolar depression + SI and caregivers of unipolar depression + no SI**

|  | | Caregivers of unipolar depression + SI | | Caregivers of unipolar depression + no SI | | Regression coefficient^b^ | *p* value^c^ |
| --- | --- | --- | --- | --- | --- | --- | --- |
|  |  | **mean ± SE** | **N** | **mean ± SE** | **N** |  |  |
| HRQoL |  |  | | | | | |
| Physical functioning | | 41.7 **±** 0.5 | 546 | 45.2 ± 0.4 | 827 | 3.45 | **<0.001** |
| Physical role functioning | | 37.5 **±** 0.4 | 546 | 40.5 ± 0.4 | 827 | 2.99 | **<0.001** |
| Bodily pain | | 39.2 **±** 0.5 | 546 | 41.6 ± 0.5 | 827 | 2.33 | **<0.001** |
| General health | | 43.5 **±** 0.5 | 546 | 43.6 ± 0.4 | 827 | 0.09 | 0.851 |
| Social role functioning | | 36.2 **±** 0.6 | 553 | 39.2 ± 0.5 | 832 | 2.97 | **<0.001** |
| Emotional role functioning | | 31.9 **±** 0.6 | 546 | 35.9 ± 0.6 | 827 | 4.03 | **<0.001** |
| Vitality | | 46.7 **±** 0.8 | 546 | 46.3 ± 0.8 | 827 | -0.45 | 0.367 |
| Mental health | | 40.2 **±** 0.6 | 546 | 41.0 ± 0.5 | 827 | 0.73 | 0.144 |
| PCS | | 42.7 ± 0.4 | 546 | 45.0 ± 0.4 | 827 | 2.30 | **<0.001** |
| MCS | | 37.5 ± 0.6 | 553 | 38.9 ± 0.6 | 832 | 1.37 | **0.007** |
| Health Status | | | |  |  | | |
| SF-6D | | 0.57 ± 0.01 | 546 | 0.60 ± 0.01 | 827 | 0.03 | **<0.001** |
| EQ-5D-5L | | 0.58 ± 0.02 | 546 | 0.66 ± 0.02 | 827 | 0.08 | **<0.001** |
| Work productivity and activity impairment (WPAI) (%) | | | | | | | |
| Absenteeism (employed only) | | 30.1 ± 6.9 | 363 | 21.9 ± 4.5 | 487 | 0.73 | 0.236 |
| Presenteeism (employed only) | | 52.9 ± 5.9 | 355 | 42.1 ± 4.1 | 478 | 0.79 | 0.105 |
| Overall work productivity loss (employed only) | | 66.4 ± 7.2 | 345 | 52.7 ± 5.0 | 470 | 0.79 | 0.093 |
| Activity Impairment, mean ± SD | | 64.9 ± 5.0 | 546 | 52.5 ± 3.3 | 827 | 0.81 | **0.026** |
| Healthcare Resource Use (past 6 months)^d^ | | | | | | | |
| HCP visits | | 10.0 ± 0.9 | 546 | 7.9 ± 0.7 | 827 | 0.79 | **<0.001** |
| ER visits | | 1.49 ± 0.29 | 546 | 0.73 ± 0.14 | 827 | 0.49 | **<0.001** |
| Hospitalizations | | 1.03 ± 0.16 | 546 | 0.52 ± 0.08 | 827 | 0.50 | **<0.001** |

*Note:* EQ-5D-5L = EuroQol 5-Dimension Health Questionnaire; HRQoL = health-related quality of life; MCS = Mental Component Score; PCS = Physical Component Score; SE = standard error; SF-6D = Short Form 6 Dimension; SF-12v2 = Revised Short Form 12 item Health Survey; SI = suicidal ideation

^a^ Valid n per model. Missing data were the result of marital status and education, or the model restriction of employed respondents.

^b^ Regression coefficients presented are β for HRQoL and health status estimates and e ^β^ for WPAI and healthcare resource use. Caregivers of unipolar depression + SI is the reference category.

^c^ Statistical testing was performed using ANOVA and statistically significant values bolded.

^d^ Multivariable models included HRU as a continuous variable. Data were available for HCP, ER and hospitalizations but not for specialists.
